# The need to increase antimicrobial resistance surveillance among forcibly displaced persons (FDPs)

**DOI:** 10.1186/s40794-023-00198-6

**Published:** 2023-09-01

**Authors:** Sodiq Inaolaji Yusuff, Yusuf Amuda Tajudeen, Iyiola Olatunji Oladunjoye, Habeebullah Jayeola Oladipo, Olufunmilayo Victoria Bolarinwa, Olalekan Tolulope Popoola, Abdulhakeem Funsho Ahmed, Matifan Dereje Olana

**Affiliations:** 1 Department of Medicine, Faculty of Clinical Sciences, Obafemi Awolowo University, Ibadan-Ife Rd, Ife, 220101 Osun State Nigeria; 2 Department of Microbiology, Faculty of Life Sciences, University of Ilorin, P.M.B. 1515, Ilorin, 240003 Nigeria; 3 Department of Epidemiology and Medical Statistics, Faculty of Public Health, College of Medicine, University of Ibadan, P.M.B 5017 G.P.O, Ibadan, Oyo State Nigeria; 4 Faculty of Pharmaceutical Sciences, University of Ilorin, P.M.B. 1515, Ilorin, 240003 Nigeria; 5 Faculty of Pharmacy, Madonna University, P.M.B 05 Elele, Rivers State Nigeria, Okija, Anambra State Nigeria; 6 Department of Public Health, Health Sciences Centre, University College, Dublin, 4 Stillorgan Rd, Belfield, Dublin 4 Ireland; 7 Faculty of Health Sciences, Department of Public Health, Al-Hikmah University, Ilorin, 240281 Kwara State Nigeria; 8 Institute of Basic and Applied Science, Department of Science Laboratory Technology, Kwara State Polytechnic, P.M.B 1375, Ilorin, Kwara State Nigeria; 9 Department of Medical Laboratory Sciences, Collage of Medicine and Health Sciences, Ambo University, Ambo, Ethiopia; 10 Department of Microbiology, Immunology and Parasitology, School of Medicine, College of Health Sciences, Addis Ababa University, P.O. Box 9086, Addis Ababa, Ethiopia

**Keywords:** Antimicrobial resistance, Antimicrobial resistance surveillance, Forcibly displace persons, Drug resistant pathogens

## Abstract

Antimicrobial resistance (AMR) poses a significant threat to human health as 4.95 million deaths were associated with bacterial AMR in 2019 and is projected to reach 10 million by 2050. To mitigate AMR, surveillance is an essential tool for determining the burden of AMR and providing the necessary information for its control. However, the global AMR surveillance is inadequate and particularly limited among forcibly displaced persons (FDPs) despite having higher risks of harboring these pathogens. Predisposing factors among this group include poor living conditions, limited access to treatment and diagnostic tests, and inadequate trained health professionals in refugee camps. Strengthening AMR surveillance among FDPs would address the identified gaps and facilitate formulation and implementation of evidence-based policies on AMR control and prevention response. This article provides information on the growing population of FDPs, factors contributing to the AMR burden and AMR surveillance gaps in FDPs and highlighted recommendations for control.

## Definition of terms

- Antimicrobial Resistance is a condition where substances particularly drugs that are capable or killing or preventing the action of microorganisms are no longer active against them [1].
- Forcibly Displaced Persons- are people that are involuntarily displaced from their home country and they include refugees, asylum seekers and others who need international protection [2]
- Asylum seekers are people who leave their country of origin in search of protection from persecution and violation of human right in another country (https://www.amnesty.org/en/what-we-do/refugees-asylum-seekers-and-migrants/#:~:text=An%20asylum%20seeker%20is%20a,asylum%20is%20a%20human%20right).
- Refugees are people that are involuntarily displaced from their country by war, violence, conflict etc. and are in search of safety in another country (https://www.amnesty.org/en/what-we-do/refugees-asylum-seekers-and-migrants/#:~:text=An%20asylum%20seeker%20is%20a,asylum%20is%20a%20human%20right)
- Migrants are people who move from one country to another especially in search for a better living condition (https://www.amnesty.org/en/what-we-do/refugees-asylum-seekers-and-migrants/#:~:text=An%20asylum%20seeker%20is%20a,asylum%20is%20a%20human%20right).
- Internally Displaced Persons are people who are forced to leave their home countries but remain within the border of their country [2].

## Introduction

The threat of antimicrobial resistance (AMR) remains alarming, as 4.95 million deaths were associated with bacterial AMR in 2019 [3]. This indicates that there is a risk of attaining the predicted annual 10 million AMR-related deaths before 2050 [4]. Intensifying efforts includes improving rapid diagnostics of infectious diseases, discovering new antibiotics & vaccines, enforcing regulations on antimicrobial stewardship and ultimately improving AMR surveillance globally [5]. Although there has been an increase in AMR surveillance in the general population over the past decade, even though not sufficient, surveillance has been particularly limited among a key population group—forcibly displaced persons (FDPs)— whose conditions predispose them to AMR [6, 7]. Recent epidemics and pandemics, such as Ebola, COVID-19, and Middle East Respiratory Syndrome (MERS), have shown that migration contribute to the spread of infectious diseases including AMR [8]. Considering that the living conditions of FDPs—poor sanitation, inadequate water supply, overcrowding, and malnutrition—provide an optimal environment for the incubation of AMR, their chance of being exposed to AMR is higher than the voluntary travelers [9]. A study on AMR amongst European migrants revealed that people subjected to forced migration and the exclusionary living conditions that often ensue had a higher prevalence of carriage or infection with AMR than other migrant groups [10]. This necessitates the need to prioritize AMR surveillance among this population to identify the right strategies to mitigate AMR burden in this group. In this article, we provide information on the growing population of FDPs, factors contributing to the AMR burden in this group, AMR surveillance gaps and recommendations to address these issues.

## Growing population of FDPs

According to the United Nations High Commissioner for Refugees (UNHCR), the number of FDPs is estimated at 90 million globally—the highest since World War II—propelled by the rising persecution, conflict, violence, human rights violations and other events actively disturbing public order, such as natural disasters. Internally-displaced people, refugees and asylum seekers account for about 57%, 29%, and 5% of the FDP population, respectively [2]. 83% of refugees and asylum seekers are hosted in low and middle-income countries, where disease surveillance is generally weak [2]. Estimations from Russian/Ukraine war revealed that around 12.8 million people have been displaced in Ukraine so far, majority of who have not left the country (internally displaced) [11] (https://guardian.ng/news/germany-records-almost-a-million-ukrainian-refugees/). Recent estimation shows that about 7.7 million people are internally displaced as a result of the conflict. Also, in Africa, an upward trend of forced displacement has been observed with over 32 million Africans displaced over the past decade largely due to repression of government against citizens, and extremist group violence (https://reliefweb.int/report/world/32-million-africans-forcibly-displaced-conflict-and-repression).

## Factors contributing to the burden of AMR among FDPs

### Endemicity of AMR in FDPs’ country of origin

A significant factor contributing to AMR burden among forced migrants is the baseline endemicity of AMR in the countries of origin [12]. Although Antimicrobial Stewardship Programs (ASPs) have been widely engaged in many High-Income Countries (HICs), the extensive adoption of hospital-based ASPs in Low- and Middle- Income Countries (LMICs) has not been accomplished [13]. This poor implementation of ASPs in LMICs has also affected countries like Syria, Venezuela, Afghanistan, South Sudan, and Myanmar where majority (69%) of refugees originate from hence, fostering their predisposition to AMR [2, 14]. For instance, studies conducted in two major Syrian cities, Aleppo and Damascus, found that over 85% of antibiotics were sold without prescription, and similar practice is seen across the Middle East, where the prevalence rate of antibiotic self-medication ranges from 19–82% [15, 16]. Due to issues facing ASPs in LMICs, it is unsurprising that 60% of wounded Syrian refugees in Germany screened for AMR harbored gram-negative Multidrug Resistance (MDR) pathogens [17].

### Unfavorable climate conditions

Issues of flood arising on account of climate change in many LMICs especially countries with the most refugees also predispose them to AMR. Climate change fosters the spread of AMR as climate warming increases the capacity of the atmosphere to hold water leading to several disasters including flooding [18]. This has the ability of contaminating domestic water with sewage containing AMR [18], hence the risk of acquiring AMR among FDPs. In addition to this, climate change across this region also results in increase in the atmospheric temperature thereby resulting in heat which favors the spread of AMR [19].

### Poor living conditions of migrants

Inadequate living conditions of migrants, such as inadequate living space, unavailability of safe water and adequate sanitation facilities, and poor access to healthcare and comprehensive case management also favors the spread of AMR [12]. For instance, the immigrant housing overcrowding rate was discovered to be 17% in the Organization for Economic Co-operation and Development (OECD) and the European Union (EU), against 8% and 11% among the native-born, respectively and about 30% of immigrants live in relative poverty in both the OECD and the EU (https://www.oecd-ilibrary.org/docserver/9789264307216-8-en.pdf?expires=1675379214&id=id&accname=guest&checksum=BE18691757242A6E6F247EAA2425C4CE#:~:text=Poverty%20adversely%20affects%20the%20well,key%20factor%20in%20well%2Dbeing). Also, limited international data available on undocumented immigrants suggest they are extremely vulnerable to lower self-reported health, accidents, injuries, psychosocial distress and unsafe water [20]. For instance, the United Nations High Commissioner for Refugees (UNHCR) ascertained that majority of refugee camps are yet to meet the international water accessibility limit (the accessibility of physically available, safe, acceptable and affordable water whose source must be within 1000 m from home and collection time must not exceed 30 min [21]), and several studies have shown water to be a major environmental reservoir of infectious diseases including AMR. Studies conducted by Hayward et al. have shown that many wastewaters which eventually find their way into water bodies contain AMR [22]. Poor infrastructure for wastewater treatment and deliberate contamination of water sources in refugees’ country of origin and camps have increased their susceptibility to AMR. Studies carried out Alhaj and Kassem revealed that poor wastewater infrastructure in Lebanon camp for Syria refugees resulted in the detection of mcr-1 in Proteus mirabilis (a gene that can confer resistance to colistin) in domestic water in the refugee camp [23]. Among the FDP groups, the refugee appears to have relatively better living conditions due to more attention from international agencies, as this brings about funding and other necessary assistance, compared to IDP camps in which the national government often needs to request for such aid before it is granted [24]. Despite the funding from the international community, many refugees still have unmet health needs [25]. For instance, a study carried out by Suphanchaimat et al. in Thailand revealed the prevalence of unmet needs among urban refugees and asylum seekers (URAS) to be 54.1% 55. Even worse is the health care of asylum seekers as the majority, particularly those without essential documents, cannot access health care due to strict regulation policies and fear of being caught, thereby falling between the cracks of health care providers and organizations providing humanitarian aids [12]. This will likely further exacerbate the health conditions of this group, with the increasing possibility of multiplying the existing AMR genes. Nellums and colleagues further demonstrated that, while transmission of AMR in FDPs’ countries of origin is more predominant due to their vulnerable circumstances, transmission of AMR still occurs during the migration trajectory in transit or the host nations [10]. Other studies [26, 27] further demonstrated this finding with migrants following similar travel paths found to be colonized with the same organism. Another cross-sectional study also showed Methicillin-Resistant Staphylococcus aureus (MRSA) clusters with transmission incidents across four refugee camps in Switzerland with no trace to migrants' origins or migratory trajectory (https://www.ecdc.europa.eu/en/about-us/networks/disease-networks-and-laboratory-networks/ears-net-about). This further emphasize that AMR is significantly acquired in the host nations or while in transit, suggesting that AMR transmission occurs between migrants or from the local population to migrants. Hence, it is necessary to investigate and curtail those factors responsible for AMR transmission at each migration phase.

#### AMR surveillance gaps among FDPs

Although there are significant gaps in AMR surveillance among FDPs globally, AMR surveillance among European refugees still relatively fares better. While there is a need for more studies among European migrants, studies among refugees and Internally Displaced Persons (IDPs) in low-income nations, where the majority of FDPs are hosted, are lacking, resulting in a dearth of data regarding AMR trends among FDPs hosted in low-income countries [28].

### AMR surveillance among FDPs in Middle and South East Asia

Generally, AMR surveillance of the populace in the Middle East Asia and Africa, where over 60% of FDPs are hosted, is lacking [2, 28]. For instance, in many region of the South Eastern Asia, implementation of the NAPs on AMR is sub-optimal (poor implementation of NAPs as studies conducted among 10 South East Asia countries by Chua et al. 2021 revealed that only parts of the NAP frameworks and not the whole, were implemented by many of the countries [29]), albeit most countries have developed their NAPs [30]. In addition to this, there is also a paucity of AMR surveillance network specific to Asia [30] and one health approach for synergizing effort on AMR surveillance is also lacking [30].

### AMR surveillance among FDPs in sub-Saharan Africa

Also, there is a paucity of studies on AMR among FDPs in sub-Saharan Africa (sSA) and this is majorly due to the absence of regional sub-Sahara African network coupled with paper-based nature of some information systems have made the collection of AMR data very demanding and time consuming [31]. For instance, a study reported that barely 11 (25%) out of 44 sSA countries had National Action Plans (NAPs) on AMR, with just 32% and 2% performing routine AMR surveillance on clinical and veterinary pathogens, respectively [32]. This is an emergency considering the high preponderance of antibiotic misuse and overuse in these regions, ranging from 4.4 to 27.3 daily doses per 1,000 inhabitants [33]. Besides, Owoaje et al. reported that over 80% of physical health symptoms in IDP camps in sSA are due to infectious diseases, predominantly malaria, acute respiratory infection and diarrhea [29]. Considering the background history of antibiotic misuse, poor living conditions of IDPs and refugee camps in this setting, inadequate numbers of trained health professionals and absence of relevant diagnostic tests to confirm the presence of specific diseases, the AMR prevalence is reasonably expected to be significant. Hence, it is unjust to leave out this population as they are more likely to succumb to the devastating effects of AMR pathogens due to the harsh conditions they live in—more than 52% and 24% of children and adults respectively living in IDPs in Africa are malnourished [29]. Weak immunity resulting from malnutrition increases the chances of infections including AMR infection and there is currently paucity of research in this area as only little attention have been given to malnutrition being a facilitator of AMR especially in sSA. A study carried by Ahmed et al. among 402 hospitalized under 5 children suffering from bacteremia in Tanzania shows the point prevalence of bacteremia among malnourished children to be 56/402, with multidrug resistant bacteria being the causative agent [34].

#### AMR surveillance in Europe

Despite the dearth of AMR surveillance among many regions, there has been a commendable effort in Europe. EARS-Net and CAESAR are major networks involved in AMR surveillance in Europe. The success of these networks in giving detailed data on AMR is due to proper networking across around 30 different European countries and 19 countries in Eastern Europe and central Asia respectively. The detailed networking and effective communication across all the regions involved, coupled with adequate funding have largely contributed to the effectiveness of AMR surveillance in Europe (https://www.ecdc.europa.eu/en/about-us/networks/disease-networks-and-laboratory-networks/ears-net-about). Both networks collaboratively provide surveillance data for almost all the 53 Member States in the WHO European Region [28] although AMR surveillance among FDPs is currently lacking. Also, Also, Nellums et al. estimated that about 23 studies on AMR among migrants were conducted between 2006 and 2016 across 7 European countries with the pooled prevalence of any detected AMR carriage or infection among FDPs (33%) higher than that of other migrants (6.6%) [10]. Another WHO community-based study on AMR among Syrian refugees and host communities in Turkey showed refugees had higher Methicillin-Resistant *Staphylococcus aureus* (MRSA) and Extended Spectrum Beta-Lactamase (ESBL) positivity rates than Turkish citizens (Refugees MRSA & ESBL: 6.7% & 17.9% respectively; Turkish citizens MRSA & ESBL: 3.2% and 14.3% respectively) [7]. These signify the preponderance of AMR among FDPs, compared to the local population. The Global AMR & Use Surveillance System (GLASS), which is the first global collaborative effort to standardize AMR surveillance aimed at providing standardized approach in the collection, analysis, interpretation and sharing of data by countries. The approach seeks to actively support capacity building and monitor the status of existing and new national surveillance systems. However, despite the increased AMR surveillance in Europe, there is need for further improvement. For instance, AMR data that eventually get into GLASS are only those approved by the country, hence the risk of being biased [31].

There is a common limitation among all AMR surveillance gaps among many regions of the world, which is the discrepancies in the stages of development of AMR surveillance systems which in turn has made cross-country comparisons impossible [31]. Hence, adequate comparison cannot be made between the prevalence of AMR in the refugees’ countries of origin and destination countries.

##### AMR surveillance gap among different regions

**Table:** 

Region	Surveillance gaps
Globally	• The discrepancies in the stages of development of AMR surveillance systems across different countries have made cross-country comparisons impossible [31]. Hence, cross country AMR data required for curbing data among FDPs are lacking
Europe	• AMR data that eventually go into GLASS are only those approved by countries, hence the risk of sample bias as it may not be an accurate representation of the country’s data [31]
Asia	• Lack of formal AMR surveillance network specific to Asia Specific Region [30], although several networks collect data on selected pathogens in this region. This region also lacks multi-sectoral and one health approach for synergizing efforts on AMR surveillance [30]
Africa	• Lack of regional sub-Sahara African network coupled with paper-based nature of some information systems have made the collection of AMR data very demanding and time consuming [31]
North America	• There is an established comprehensive surveillance system for AMR in United States and Canada. However, there is a need for better coordination and data sharing between different states and provinces. Additionally, surveillance gaps might exist in less populated areas or among certain healthcare facilities with limited resources [35]
South America	• In this region, AMR surveillance systems countries. Countries such as Brazil, Argentina, and Colombia, have made significant progress in establishing surveillance networks and implementing national action plans. However, other countries in the region such as Bolivia, El Salvador, and Honduras face challenges due to limited resources, infrastructure, and coordination among healthcare systems [36]
Australia/Pacific	• There is a robust surveillance system for AMR in Australia through the Australian Group on Antimicrobial Resistance (AGAR). The country has implemented various initiatives to monitor and respond to AMR. In the Pacific region, surveillance capacities vary among individual countries, with some facing resource constraints and limited infrastructure for comprehensive AMR surveillance [36]

## Recommendations and conclusion

To address the issues above, the following recommendations are required:

Overall strengthening of AMR surveillance globally especially in low income countries: There is a need for overall strengthening of AMR surveillance globally, particularly in low-income nations. For instance, upper middle- and high-income nations have a central repository for collecting AMR data, such as Central Asian and European Surveillance of Antimicrobial Resistance (CAESAR), the European Antimicrobial Resistance Surveillance Network (EARS-Net), the Latin American Network for Antimicrobial Resistance Surveillance (Rede Latinoamericana de Vigilancia de la Resistencia a los Antimicrobianos [ReLAVRA]), and the Western Pacific Regional Antimicrobial Consumption Surveillance System (WPRACSS) (https://www.oecd-ilibrary.org/docserver/9789264307216-8-en.pdf?expires=1675379214&id=id&accname=guest&checksum=BE18691757242A6E6F247EAA2425C4CE#:~:text=Poverty%20adversely%20affects%20the%20well,key%20factor%20in%20well%2Dbeing). Unfortunately, such regional AMR networks are lacking in sSA, thereby limiting the availability of relevant AMR data from the continent. In order to rectify this issue, there is the need for the creation of central repository for the collection of AMR data, proper coordination of this repository by health experts and other required professionals and so on.

- Ensuring strong regional network in sub-Saharan African countries

Regional level data on AMR and FDPs is important as this will inform the required strategy and the accurate amount of resources required for curbing the spread of AMR. Data on AMR in FDPs’ region of origin will alert refugee camp coordinators on imposing necessary measures that will curb the spread of AMR. Also, a regional sSA network would bridge data gap on FDPs because it will help in tracking the specific point of acquisition of AMR (whether region of origin or in transit), making accurate data on FDPs available.

- Incorporation of well spelt-out strategies for curbing AMR into GLASS and NAPs.

It is also worth noting that the WHO Global Actional Plan on AMR and Global AMR & Use Surveillance System (GLASS) do not have specific strategies for conducting AMR surveillance among FDPs, thereby necessitating the revision of these policy documents. Likewise, each country's AMR National Action Plans should clearly articulate the strategies to improve AMR surveillance among refugees and IDPs. Some of these strategies include, increasing AMR surveillance in countries of AMR prevalence, prioritizing AMR surveillance during transit, incorporating AMR surveillance on refugee camps, enforcing measures that prevent the spread of AMR in refugee camp where detected and provision of conducive environment and maximum resources for refugees.

- Speedy addressing of factors preventing the implementation of NAPs

Also, factors hindering the adequate implementation of NAPs, as elucidated by Kariuki et al., such as lack of political commitment, inadequate funding, and inadequate capacity [37], need to be swiftly addressed by conducting strategic advocacy to the policymakers to increase funding for AMR surveillance. Another key step in the right direction is determining the baseline AMR of displaced people and AMR level in transit and while in the host nations. This would perhaps help understand the full picture of AMR transmission dynamics among migrants, as each migration stage has its peculiarities and might require different measures to curtail the transmission of AMR. Of recent, the German government authorized MDR organism screening of refugees upon admission to healthcare facilities [38]. However, this practice should be recommended at the early stage of registering refugees in order to know the baseline endemicity of AMR among displaced people. Also, as evidence has shown that living conditions of the IDP and refugee camps provide optimal conditions for AMR to thrive, a robust surveillance system would help in identifying which areas contribute the most to the AMR burden, leading to advocacy to policymakers to prioritize addressing those areas, as resources are finite. Ultimately, there would be a need to improve the overall living conditions of FDPs by providing adequate water supply, proper sanitation, and good hygiene to deter AMR incubation and also allow FDPs, particularly asylum seekers, to access good health care regardless of their status as it is their human rights.

## Total regulation of antimicrobial stewardship

Total ban on unethical administration of antimicrobials will go a long a way in curbing the burden of AMR. This involve enforcing policies that allow only certified personnels to administer antimicrobials. Also, more sensitizations on the risk of antimicrobials should be carried out especially in sub-Sahara Africa countries where unethical administration and use of antimicrobials is more common.

## Data Availability

Not applicable.

## Abbreviations

AMR: Antimicrobial resistance
MDR: Multidrug resistance
MERS: Middle East Respiratory Syndrome
UNHCR: United Nations High Commissioner for Refugees
MRSA: Methicillin-Resistant Staphylococcus aureus
ESBL: Extended Spectrum Betalactamase
sSA: Sub-Sahara Africa
ASPs: Antimicrobial Stewardship Programs
LMICs: Low- and Middle- Income Countries
